# Gab3 overexpression in human glioma mediates Akt activation and tumor cell proliferation

**DOI:** 10.1371/journal.pone.0173473

**Published:** 2017-03-14

**Authors:** Pifeng Jia, Feng Li, Weiting Gu, Weifeng Zhang, Yu Cai

**Affiliations:** Department of Neurosurgery, RuiJin Hospital North, Shanghai Jiao Tong University School of Medicine, Shanghai, China; University of South Alabama Mitchell Cancer Institute, UNITED STATES

## Abstract

This current study tested expression and potential biological functions of Gab3 in human glioma. *Gab3* mRNA and protein expression was significantly elevated in human glioma tissues and glioma cells. Its level was however low in normal brain tissues and primary human astrocytes. In both established (U251MG cell line) and primary human glioma cells, Gab3 knockdown by shRNA/siRNA significantly inhibited Akt activation and cell proliferation. Reversely, forced Gab3 overexpression in U251MG cells promoted Akt activation and cell proliferation. *In vivo*, the growth of U251MG tumors in nude mice was inhibited following expressing Gab3 shRNA. Akt activation in cancer tissues was also suppressed by Gab3 shRNA. Together, we conclude that Gab3 overexpression in human glioma mediates Akt activation and cancer cell proliferation.

## Introduction

Glioma is a common cancer in the central nerves system (CNS), which causes significant human mortalities each year [1,2,3]. The prognosis of high-grade glioma (grade III-IV) is extremely poor [1,2,3]. The incidence of this devastating disease, however, has been rising in certain areas [1,2,3]. The application of current clinical treatments, including postoperative radiation and/or adjuvant temozolomide, failed to significantly improve patents’ five-year survival[4,5,6]. One possible factor could be the molecular heterogeneity of glioma [1]. Therefore, our group has been dedicated to indentify novel oncogenic proteins for human glioma [7,8,9,10,11].

The mammalian Gab [growth factor receptor binding 2 (Grb2)-associated binding protein] family of proteins have three members, Gab1, Gab2 and Gab3 [12,13]. The adaptor protein Gab3, like other Gab proteins, could be tyrosine phosphorylated [14,15,16]. Gab3 associates with the SH2 domain-containing proteins, *i*.*e*. PI3K regulatory subunit p85 [14,15,16]. This presumably will mediate Akt signaling activation [14,15,16]. Existing evidence has demonstrated that Gab3 is more restricted to the hematopoietic tissues (*i*.*e*. spleen and thymus) [14,15,16]. Yet, its expression is also detected in other tissues, including heart, kidney, uterus, and brain [14,15,16]. A very recent study has explored expression of Gab1 in human glioma [17]. To our best knowledge, the expression and potential biological functions of Gab3 in human glioma have not been evaluated. Here, our preliminary results suggest that Gab3 overexpression in human glioma mediates Akt activation and cancer cell proliferation.

## Materials and methods

### Chemicals and reagents

Antibodies for Gab1 (#3232), p-Akt (Thr 308, #9275), Akt1/2 (9272), (β-) tubulin (#2128) p-p44/42 MAPK (p-Erk1/2, #9101), Erk1/2 (#9102), SHP2 (#3752), PI3K subunit p85 (#4292) were purchased from Cell Signaling Tech (Shanghai, China). The anti-Gab3 (sc-271475) antibody was obtained from Santa Cruz Biotech (Shanghai, China). Perifosine (S1037) and LY294002 (S1105) were obtained from Selleck (Shanghai, China). Annexin V-FITC apoptosis detection kit was obtained from Abcam (ab14085, Shanghai, China).

### Culture of established cells

The culture of established human glioma cell lines, U251MG, as well as culture of primary human astrocytes were described previously [7,8,9,10,11].The protocols of culture of primary human astrocytes were approved by the Ethics Review Board (ERB, approval number SJD-2014-55) of Shanghai Jiao-Tong University School of Medicine (Shanghai, China).

### Acquirement of human glioma tissues

Surgery-isolated high-grade (III-IV) human glioma tissues along with paired surrounding normal tissues were homogenized and lysed, expression of listed genes was tested by Western blotting assay and/or PCR assay. A total of seven glioma patients, hospitalized at Rui-Jin Hospital, Shanghai Jiao-Tong University School of Medicine (Shanghai, China), were consent to provide specimens. These patients received no prior treatment before surgery. The written-informed consent was obtained from the each enrolled patient. The protocols requiring human specimens were approved by the ERB (approval number SJD-2014-102) of Shanghai Jiao-Tong University School of Medicine. All clinical investigations were conducted according to the Code of Ethics of the World Medical Association (Declaration of Helsinki). The privacy rights of human subjects are always observed.

### Primary culture of human glioma cells

The protocol was described early [17]. In brief, acquired fresh human glioma tissue from one patient was thoroughly washed in PBS and DMEM, which was then minced and filtered. Single-cell suspensions were achieved by digestion in Collagenase I (Sigma) for 30 min in DMEM at room temperature [17]. Primary human glioma cells were cultured in the FBS (16000–044, Gibco)-DMEM/F12 medium (21700–075, Gibco), plus 5 ng/mL basic fibroblast growth factor (bFGF, F5392, Sigma) and 10 ng/mL epidermal growth factor (EGF, E5036, Sigma).The protocols using primary human glioma cells were again approved by the ERB (approval number, SJD-2014-135) of Shanghai Jiao-Tong University School of Medicine.

### Cell MTT assay

The cell proliferation was tested by the routine MTT assay, the detailed protocol is described in our previous studies [7,8,9,10,11].

### Cell death detection

Cells with applied treatment were harvested, washed and stained with trypan blue dye (Sigma). Cells with compromised cell membranes took up trypan blue, and were counted as dead cells [8,9].

### BrdU assay of proliferation

Cells with the designated treatment were incubated with BrdU (5 μM) and were fixed. BrdU incorporation, deterring cell proliferation, was tested by the BrdU ELISA kit (Roche Diagnostics).

### Clonogenicity assay

As described, [8,9,18], U251MG cells with applied genetic modification (5 × 10^3^ per dish) were resuspended in agar-containing medium, and were cultured on a 100 mm culture dish. After 10 days of culture, the proliferative colonies were manually counted.

### Apoptosis analysis

As described early [9],cells with applied treatment were washed. Afterwards, propidium iodide (50 μg/mL), Annexin V-FITC (50μg/mL) and 0.1% Triton X-100 were added [8]. Annexin V positive cells (apoptotic cells) were gated by FACS machine (FACS Canto II flow cytometer, Shanghai, China).

### Quantitative real-time reverse transcriptase polymerase chain reaction (qRT-PCR assay)

RNA was extracted via the routine Trizol reagents (Invitrogen). For each preparation, 1 μg RNA, 100 nM primers and 1× SYBR Master Mix (Applied Biosystem, Shanghai, China) were mixed. The ABI Prism 7600 Fast Real-Time PCR system (Foster City, CA) was applied for real-time PCR reactions. Melt curve analysis was tested to analyze product melting temperature. Quantification of listed mRNA was through the ^ΔΔ^Ct method. *GAPDH* was also tested as the internal control. The primers were as follows:Gab1: forward, 5’-GCATGGAAGAGGAGATGGTTCGTGT-3’ and reverse: 5’-AGTAGCAGAGGATGAATCTGCCTGG-3’[19]; Gab3, forward: 5’-GAGAGCCTCTCTTACACG-3’ and reverse: 5’-GGCTGAAGCTGTGGGGTA-3’ [19]; GAPDH, forward: 5’-TGAAGGTCGGAGTCAACGGATTTGG-3’ and reverse: 5’-CATGTGGGCCATGAGGTCCACCAC-3’ [19]. The primers were purchased from Jikai (Suzhou, China).

### Western blotting assay

As described [9], equal amount of lysates (30 μg per sample) were separated by SDS-PAGE gels, and were transferred to PVDF membranes. After blocking, indicated primary antibody and secondary antibody were added. Enhanced chemiluminescence (ECL) reagents (Amersham, Shanghai, China) were applied to visualize the intend band. The densitometry of each band was quantified via the ImageJ software, and its value was always normalized to the corresponding loading control(unless otherwise noted)[9].

### Immunoprecipitation (IP)

The detailed protocol was described in other studies [20,21]. Briefly, aliquots of 800 μg of protein lysates from each sample were pre-cleared by protein A/G beads (Sigma), followed by incubation with anti-Gab3 antibody overnight. Thirty μl of protein A/G beads (Sigma) were then added, and the lysates were incubated for 2 hours at 4°C. The beads were washed with PBS for 5 times. Afterwards, Gab3-assocaited proteins were detected by Western blotting assay.

### Gab3 knockdown by shRNA and stable cell selection

The three different lentiviral Gab3short hairpin RNAs (“shGab3-a/-b/-c”, Catalog no. GP-2015-139716sh-a/-b-c) were designed by Jikai Biotech (Suzhou, China). The shRNA was added to cultured U251MG cells for 24 hours. Afterwards, puromycin (0.5 μg/mL, Sigma) was applied to select stable clones for 8–9 days. Control cells were incubated with lentiviral scramble non-sense shRNA (Santa Cruz) [9,18]. Gab3 knockdown in stable cells was verified by Western blotting assay and/or qRT-PCR assay.

### Gab3 RNA interference in primary cells

Two Gab3 siRNAs (Gab3 siRNA-a: from Santa Cruzsc-40608, Gab3 siRNA-b: from ABM,i008390) and a non-specific scramble siRNA (“si-SCR”, Santa Cruz) were applied, and transfected into primary human glioma cells with Lipofectamine 2000 (Invitrogen). Knockdown efficiency was again determined by qRT-PCR andWestern blotting assay 48 hours after transfection.

### Gab3 overexpression

The human Gab3 cDNA, synthesized by theJikai Biotech (Shanghai, China), was inserted into the GV428 expression vector, which contained a Flag tag and a puromycin resistance gene. U251MG cells were seeded onto six-well plates. Gab3 expression construct (0.25 μg/mL) was transfected to U251MG cells via the Lipofectamine 2000 reagents [22], and stable cells were selected by the puromycin. Transfection efficiency was verified via testing Gab3 (Flag-tagged) expression in stable cells.

### Xenograft assay

The female nude mice (4–5 week-old, 19–20 g in weight) were purchased from the Animal Center of Shanghai Jiao Tong University School of Medicine (Shanghai, China). Mice were fed with an autoclaved laboratory rodent diet. U251MG cells (10 × 10^6^ cells in 200 μl of Matrigel per mouse), with scramble control shRNA or Gab3 shRNA (-c), were inoculated (*s*.*c*.) to the right flanks of the nude mice. Within three weeks, the tumor volume reached around 100 mm^3^, and the recordings were initiated. The tumor volumes and mice body weights were recorded every 6 days. Volumes were calculated via the formula: π/6×width ^2^× length. Estimated average daily tumor growth was also calculated [22,23]. The animal protocols were approved by Institutional Animal Care and Use Committee (IACUC) and ERB (approval number, BR-2014-188) of Shanghai Jiao-Tong University School of Medicine (Shanghai, China). Animals were observed on daily bases. Humane endpoints were defined as a loss of more that 15% of body mass, a tumor with the diameter over 1.3 cm, or inability to ambulate or rise for food and water. If reaching these endpoints, CO_2_ inhalation was used for euthanasia of animals. All mouse surgical procedures were performed with anesthesia by injection of ketamin/xylazine HCl (50 mg/kg/10 mg/kg, *i*.*p*.). All efforts were made to minimize suffering.

### Statistical analysis

Statistics were performed through the SPSS 18.0 software (Chicago, IL). For each set of *in vitro* cell line experiment, at least 5 replicate wells (n = 5) were included, mean ± standard deviation (SD) were calculated. Statistics was then performed. Afterwards, the whole set of experiment was repeated 3–5 times, making sure similar results were reproduced. p<0.05 was considered statistically significant.

## Results

### Gab3 overexpression in both human glioma tissues and glioma cells

**Fig 1:**
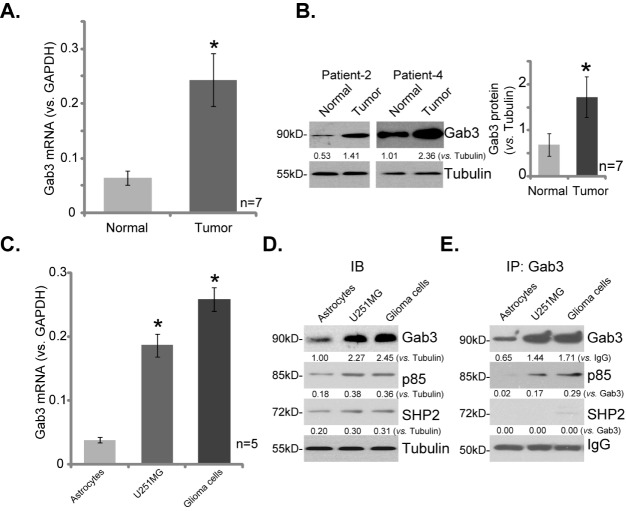
Gab3 overexpression in human glioma tissues and glioma cells. *Gab3* mRNA and protein expression in described tissues and cell lines were detected by qRT-PCR assay (A and C) and Western blotting assay (B and D), respectively. SHP2 and p85 expressions were also tested (D). Gab3 association with p85 or SHP2 was tested by immunoprecipitation (IP) assay (E). (A and B) n = 7 means seven sets of tissues. (C) n = 5 means five replicate wells. The intensity of each band was normalized to the corresponding loading control or indicated protein. Data were presented as mean ± SD. * p <0.05 vs. “Normal” tissues/“Astrocytes”.

We first tested expression of Gab3 in human glioma tissues. As discussed, a total of seven different high-grade glioma tissues (“Tumor”) and the paired surrounding normal brain tissues (“Normal”) were acquired. qRT-PCR assay results demonstrated that *Gab3* mRNA expression in the tumor tissues was significantly higher than that in the normal brain tissues (Fig 1A). *Gab3* mRNA level was about four times higher in tumor tissues (Fig 1A). Further analysis showed that Gab3 protein expression was also upregulated in the tumor tissues (See representative blots of two patients in Fig 1B, Left panel). Its expression in normal brain tissues was low (Fig 1B). The quantified blot results of all seven sets of tissues were integrated (Fig 1B, right panel).Gab3 expression in cultured human glioma cells was also tested. In both established (U251MG cell line) and primary human glioma cells (“Glioma cells”),*Gab3* mRNA (Fig 1C) and protein(Fig 1D) expression level was significantly higher than that in the primary human astrocytes (“Astrocytes”). Thus, these results demonstrate Gab3 overexpression in both human glioma tissues and glioma cells. Expressions of two other adaptor proteins, including p85 and SHP2, were also upregulated in the glioma cells (Fig 1D).

Next, the immunoprecipitation (IP) assay was performed to examine the association between Gab3 and other proposed adaptor proteins. Results in Fig 1E showed that there was a strong association between Gab3 and p85 in both U251MG cells and primary human glioma cells. Gab3-p85 association was not observed in the primary astrocytes (Fig 1E). Interestingly, Gab3 didn’t form a complex with SHP2 in above glioma cells (Fig 1E). These results imply that Gab3 possibly serves as the upstream of p85-Akt signaling in glioma cells.

### Gab3 knockdown by shRNA inhibits U251MG glioma cell proliferation

**Fig 2:**
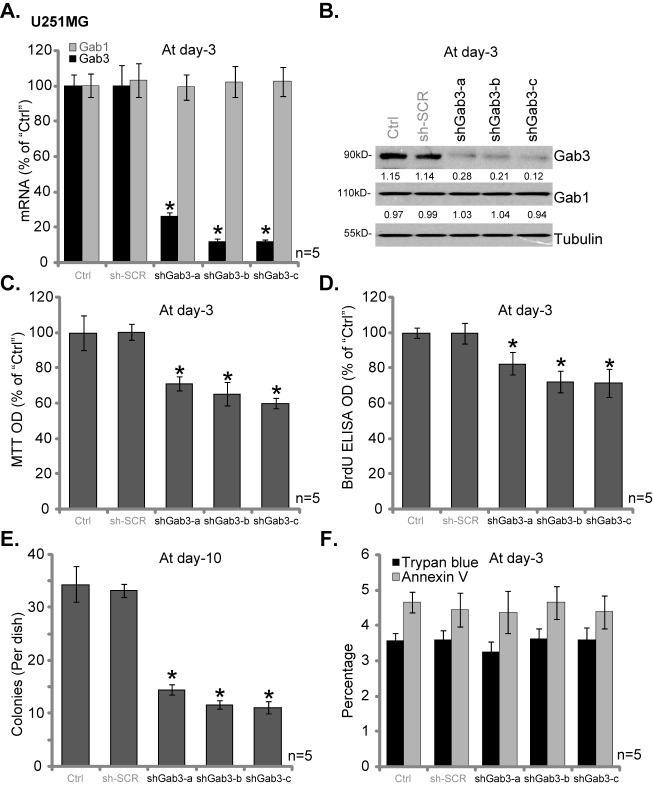
Gab3 knockdown by shRNA inhibit U251MG glioma cell proliferation. Stable U251MG glioma cells, expressing Gab3 shRNAs (“shGab3-a/-b/-c”) or scramble control shRNA (“sh-SCR”), as well as the parental control (“Ctrl”) U251MG cells, were subjected to qRT-PCR assay (A) and Western blotting assay (B) of listed genes; Cell proliferation was tested by MTT assay (C), BrdU ELISA assay (D) and Clonogenicity assay (E); Cell death (trypan blue staining assay) and apoptosis (Annexin V FACS assay) were also tested (F). Relative expression of listed proteins was quantified (vs. Tubulin, B). For all these assays, the exact same number of cells of different backgrounds were initially plated into each well/dish. Data were presented as mean ± SD. * p <0.05 *vs*. “Ctrl” cells.

In order to study the potential effect of Gab3 on human glioma cells *in vitro*, shRNA strategy was applied. As discussed, a set of three distinct Gab3 shRNAs (“shGab3-a/-b/-c”) with non-overlapping sequences were introduced to U251MG glioma cells. Puromycin was added afterwards to select stable cells. qRT-PCR assay results in Fig 2A demonstrated that each of the three applied Gab3 shRNAs dramatically downregulated *Gab3* mRNA expression in U251MG cells. *Gab1* mRNA expression was unchanged (Fig 2A). Western blotting assay results in Fig 2B confirmed Gab3 protein knockdownin above U251MG cells. Once again, Gab1 protein expression was not changed (Fig 2B). Remarkably, the proliferation of U251MG cells, tested by MTT assay (Fig 2C) and BrdU ELISA assay (Fig 2D), was inhibited significantly with Gab3 knockdown. The MTT OD and BrdU ELISA OD were both decreased in cells with Gab3 shRNAs, as compared to those of parental control (“Ctrl”) cells (Fig 2C and 2D). Meanwhile, the number of proliferative U251MG colonies was also decreased with Gab3 shRNA (Fig 2E). Intriguingly, U251MG cell death (tested by trypan blue staining assay) and apoptosis (tested by the Annexin V FACS assay) were not significantly altered after Gab3 knockdown (Fig 2F). There results suggest that Gab3 knockdown inhibits U251MG glioma cell proliferation. As expected, the scramble control shRNA (“sh-SCR”) didn’t affect Gab1/3 expression (Fig 2A and 2B) and U251MG cell proliferation(Fig 2C–2F).

### Gab3 siRNA inhibits primary human glioma cell proliferation

**Fig 3:**
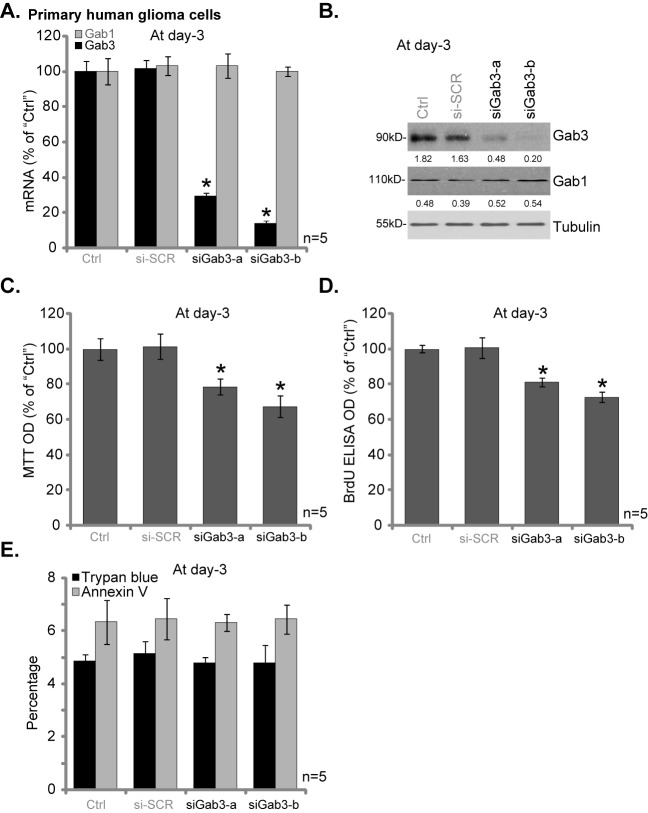
Gab3 siRNA inhibits primary human glioma cell proliferation. The primary human glioma cells, transfected with Gab3 siRNAs (“siGab3-a/-b”) or scramble control siRNA (“si-SCR”), as well as the non-transfected control (“Ctrl”) cells, were subjected to qRT-PCR assay (A) and Western blotting assay (B) to tested listed genes; Cell proliferation was tested by MTT assay (C) and BrdU ELISA assay (D); Cell death (trypan blue staining assay) and apoptosis (Annexin V staining assay) were also tested (E). Relative expression of listed proteins was quantified (*vs*. Tubulin, B). For all these assays, the exact same number of cells of different treatment were initially plated into each well/dish. Data were presented as mean ± SD. * p <0.05 *vs*. “Ctrl” cells.

Next, we tested the potential effect of Gab3 on primary cancer cells. As discussed, one primary human glioma cell line was established. siRNA strategy was utilized to transiently knockdown Gab3 in the primary cells. As shown in Fig 3A, transfection of two applied Gab3 siRNAs (“siGab3-a/-b”) efficiently downregulated *Gab3* mRNA (but not Gab1) in the primary glioma cells. Further, Gab3 protein expression was also decreased (Fig 3B). Significantly, MTT assay results in Fig 3C and BrdU ELISA results in Fig 3D demonstrated that Gab3 siRNA similarly inhibited proliferation of the primary human glioma cells. Notably, siGab3-b was more efficient in downregulating Gab3 than siGab3-a (Fig 3A and 3B), it was also more potent in inhibiting cell proliferation (Fig 3C and 3D). We failed to detect significant cell death and apoptosis following Gab3 knockdown in the primary cancer cells (Fig 3E). The scramble control siRNA (“si-SCR”) didn’t affect Gab3 expression (Fig 3A and 3B) and cancer cell proliferation (Fig 3C and 3D). Thus, knockdown of Gab3 inhibits proliferation of the primary human glioma cells. Similar results were also obtained from other replicate primary glioma cell lines (Data not shown).

### Forced overexpression of Gab3 promotes U251MG glioma cell proliferation

**Fig 4:**
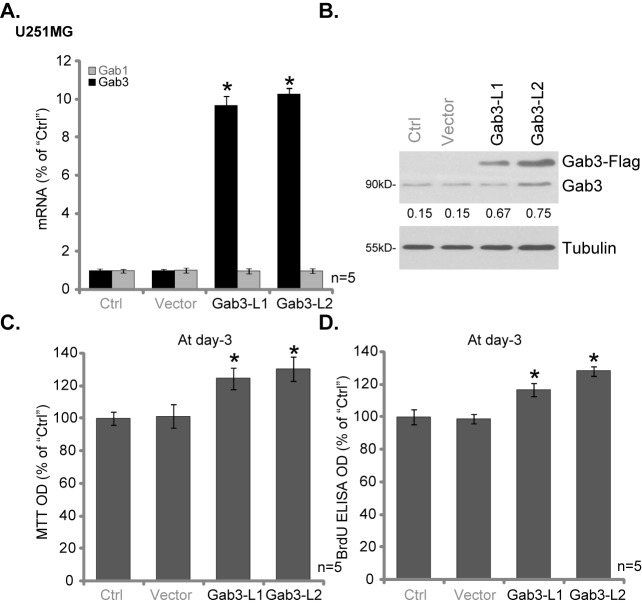
Forced overexpression of Gab3 promotes U251MG glioma cell proliferation. Stable U251MG glioma cells, expressing wt-Gab3 (two lines, “Gab3-L1/L2”) or empty vector (“Vector”, pSuper-puro), as well as the parental control (“Ctrl”) cells, were subjected to qRT-PCR assay (A) and Western blotting assay (B) of listed genes; Cell proliferation was tested by MTT assay (C) and BrdU ELISA assay (D). Relative expression of Gab3 was quantified (*vs*. Tubulin, B). For all these assays, the exact same number of cells of different treatment were initially plated into each well/dish. Data were presented as mean ± SD. * p <0.05 *vs*. “Ctrl” cells.

Next, a Gab3 expression construct was established, which was transfected to U251MG cells. Via puromycin selection, two stable U251MG cell lines-overexpressing Gab3 (Flag-tagged) were established, named “Gab3-L1/L2”. As compared to parental control (“Ctrl”) cells, mRNA (Fig 4A) and protein (Fig 4B) expression of Gab3 was significantly upregulated in U251MG cells with the construct. Gab1 expression was again unchanged in these cells (Fig 4A). Importantly, U251MG cell proliferation, tested by MTT assay (Fig 4C) and BrdU ELISA assay (Fig 4D), was augmented in Gab3-overexpressed cells (“Gab3-L1/L2”). Expectably, the empty vector (“Vector”, pSuper-puro) failed to affect Gab3 expression (Fig 4A and 4B) and U251MG cell proliferation (Fig 4C and 4D). Therefore, these results suggest that forced expression of Gab3 promotes U251MG glioma cell proliferation, further confirming a role of Gab3 in promoting glioma cell proliferation.

### Gab3 is important for Akt activation in human glioma cells

**Fig 5:**
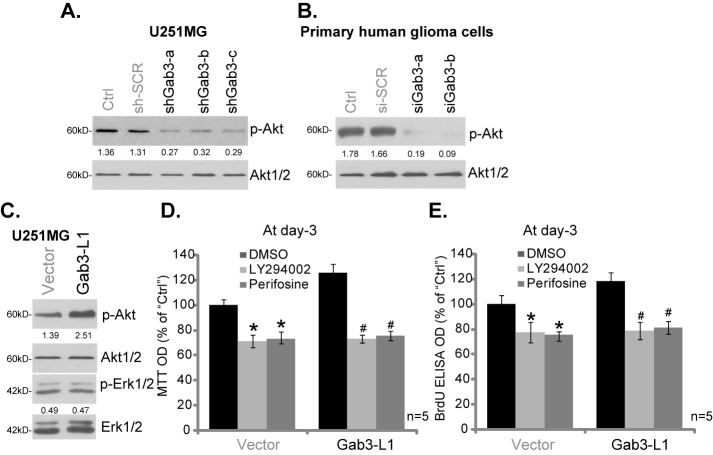
Gab3 is important for Akt activation in human glioma cells. U251MG glioma cells (A and C) or primary human glioma cells (B), with/out the listed Gab3 genetic manipulation, were subjected to Western blotting assay of listed proteins. p-Akt (*vs*. total Akt1/2) and p-Erk1/2 (*vs*. total Erk1/2) were quantified. Stable U251MG glioma cells, expressing wt-Gab3 (two lines, “Gab3-L1”) or empty vector (“Vector”, pSuper-puro), were treated with LY294002 (5 μM) or perifosine (5 μM), cell proliferation was tested by MTT assay (D) and BrdU ELISA assay (E).“DMSO” stands for 0.1% of DMSO.* p <0.05 *vs*. DMSO of “Vector” cells. ^#^ p <0.05 *vs*. DMSO of “Gab3-L1”cells.

Existing evidences have demonstrated that Akt signaling plays a pivotal role in promoting glioma cell progression [24,25,26]. Activation of Akt in glioma cells participates in a number of key cancerous behaviors, including cell proliferation, migration and motility, survival, angiogenesis and apoptosis resistance [24,25,26]. We thus tested Akt activation in above glioma cells. As shown in Fig 5A, Gab3 known by targeted-shRNA in U251MG cells dramatically inhibited Akt activation, the latter was tested by phosphorylation (p-) of Akt at Thr-308. p-Akt level was reduced in all three U251MG cell lines expressing Gab3 shRNA (Fig 5A). In the primary human glioma cells, siRNA-mediated knockdown of Gab3 also inhibited Akt activation (Fig 5B). On the other hand, as shown in Fig 5C, forced overexpression of Gab3 in U251MG cells augmented Akt activation. Gab3 overexpression failed to affect Erk1/2 activation (p-Erk1/2) in U251MG cells (Fig 5C). These results suggest that Gab3is important for Akt activation in human glioma cells.

In order to confirm the role of Akt activation in Gab3-promoted cell proliferation, Akt inhibitors were applied, including the pan PI3K-Akt inhibitor LY294002[27] and the Akt specific inhibitor perifosine[28]. Expectably, application of both inhibitors suppressed U251MG cell proliferation, which was again tested by the MTT assay (Fig 5D) and BrdU ELISA assay (Fig 5E). More importantly, Gab3 overexpression-promoted U251MG cell proliferation was almost completely blocked with treatment of the PI3K-Akt inhibitors (Fig 5D and 5E). These results suggest that Gab3 overexpression-induced glioma cell proliferation likely is mediated through activating downstream Akt.

### Gab3 knockdown suppresses U251MG xenograft tumor growth in nude mice

**Fig 6:**
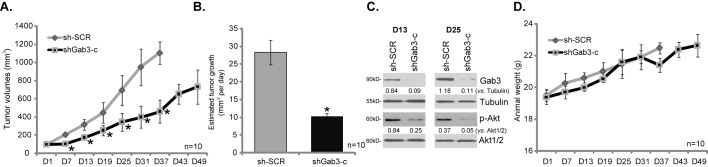
Gab3 knockdown suppresses U251MG xenograft tumor growth in nude mice. The exact same amount of stable U251MG cells (1* 10^7^ cells per mouse), expressing scramble control shRNA (“sh-SCR”) or Gab3 shRNA (shGab3-c), were inoculated *s*.*c*. into the flanks of mice to establish U251MG xenografts. Tumor volumes (A) and mice body weights (D) were recorded every 6 days; Estimated daily tumor growth was calculated (B); At Day-13 and Day-25, one U251MG tumor per group was isolated, expression of the listed protein in the fresh tumor lysates was tested by Western blotting assay (C), relative expression of listed proteins was quantified (C, *vs*. Akt1/2 or Tubulin). * p <0.05 *vs*. “sh-SCR” tumors.

In order to test the potential effect of Gab3 on glioma cell proliferation *in vivo*. A nude mice U251MG xenograft model was applied. As described, same amount of U251MG cells expressing scramble control shRNA (“sh-SCR”) or Gab3 shRNA (shGab3-c) were inoculated. Fig 6A recorded tumor volumes, and results showed that the growth of U251MG tumors with Gab3 shRNA was much slower than those with scramble control shRNA (Fig 6A). The volume of tumors expressing Gab3 shRNA was significantly lower than the control shRNA tumors (Fig 6A). When calculating daily tumor growth (in mm^3^ per day), we showed again that growth of U251MG tumor *in vivo* was dramatically suppressed following expressing Gab3 shRNA (Fig 6B). At Day-13 and Day-25, one U251MG tumor per group was isolated. Western blotting assay of tumor lysates showed that Gab3 was indeed depleted in Gab3 shRNA-expressing tumors (Fig 6C). Meanwhile, Akt activation, tested by p-Akt (Thr-308), was also reduced in the tumors (Fig 6C). Therefore, in line with the *in vitro* signaling findings, Gab3 knockdown also inhibited Akt activation *in vivo*. Results in Fig 6D demonstrated that mice body weight was not significantly different between the two groups. Together, these results show that U251MG tumor growth in mice is inhibited following expressing Gab3 shRNA, supporting a role of Gab3 on U251MG tumor growth *in vivo*.

## Discussion

In various cell types, Gab family members, including Gab1, Gab2 and Gab3, play crucial roles in mediating multiple signaling pathways, which are important for cell proliferation, survival, and differentiation [12,13,29]. Upon activation, Gab proteins are tyrosine phosphorylated, which then bind to SHP2, the PI3K subunit p85, as well as Crk family proteins. This would lead to activation of MAPK, PI3K-Akt, and JNK[12,13,29], which are all vital oncogenic pathways. Recent studies have focused on the potential function of Gab family proteins in tumorigenesis, cancer differentiation and progression [12].

To data, Gab1 is the most predominant Gab family member protein, which is ubiquitously expressed in almost all mammalian cells and tissues [12]. Studies have implied Gab1 as a oncogenic protein in multiple human cancers, whose overexpression is associated with cancer cell progression [12]. A very recent study has suggested that Gab1 is important for activation of PI3K-Akt-mTOR activation in human glioma cells [17]. On the other hand, Gab3 is the least ubiquitously expressed member of the Gab family protein, and it is predominantly expressed in hematopoietic cells [14,15,16]. In the brain, yet weak Gab3 expression was still detected [14,15,16]. To our best knowledge, the expression and potential function of Gab3 in human cancer has not been studied thus far.

It should be noted that exogenous Gab3 overexpression only increased glioma cell proliferation by 20–30% (Fig 4). This could be due to the fact that basal Gab3 expression is already high in glioma cells (Fig 1). It is also possible that other Gab proteins, including Gab1 and Gab2, could also be required for cell proliferation as well. Indeed, the recent paper by Li *et al*., has implied a role of Gab1 in glioma cell proliferation[17]. Further studies will be need to dissect the role of each Gab protein in mediating Akt activation and glioma cell proliferation. One other reason could be that Gab3 appears not required for Erk activation in glioma cells. Gab3 didn’t form a complex with SHP2 in the glioma cells. Further, exogenous Gab3 overexpression failed to affect Erk1/2 activation (p-Erk1/2) in U251MG cells.

The preclinical results of our study suggest that Gab3 should be a novel oncogenic protein for human glioma. First, Gab3 mRNA and protein expression was significantly elevated in both human glioma tissues and glioma cells, yet its level was quite low in normal brain tissues and human astrocytes. Second, glioma cell growth was inhibited by Gab3 knockdown, but was augmented with Gab3 overexpression. Third, in human glioma cells, Gab3 is required for the activation of Akt, which is key oncogenic pathway in glioma [30,31]. Importantly, growth of U251MG tumor *in vivo* was remarkably inhibited when expressing Gab3 shRNA. Therefore, Gab3 could be a novel oncotarget protein for glioma.

## Conclusion

Even with the development of modern clinical practice, the prognosis of high-grade glioma is still extremely poor, and the five-year survival is rare[3]. Late diagnosis, absence of specific markers, resistance of traditional therapy, as well as high invasion potential are all possible causes of its high-malignant nature [6,25]. Our results showing that Gab3 overexpression in human glioma mediates Akt activation and cancer cell proliferation suggest that Gab3 could be a novel oncogenic protein for glioma.
